# Case-Control Study of Vitamin D, *dickkopf homolog 1* (*DKK1*) Gene Methylation, *VDR* Gene Polymorphism and the Risk of Colon Adenoma in African Americans

**DOI:** 10.1371/journal.pone.0025314

**Published:** 2011-10-13

**Authors:** Hassan Ashktorab, Bijou Nguza, Mehrnaz Fatemi, Mehdi Nouraie, Duane T. Smoot, Alejandro A. Schäffer, Sonia S. Kupfer, Carlos A. Camargo, Hassan Brim

**Affiliations:** 1 Department of Medicine and Cancer Center, College of Medicine, Howard University, Washington, D. C., United States of America; 2 Center for Sickle Cell Disease, College of Medicine, Howard University, Washington, D. C., United States of America; 3 Department of Pathology, College of Medicine, Howard University, Washington, D. C., United States of America; 4 National Center for Biotechnology Information, National Institutes of Health, Department of Health and Human Services, Bethesda, Maryland, United States of America; 5 Section of Gastroenterology, Department of Medicine, University of Chicago, Chicago, Illinois, United States of America; 6 Department of Emergency Medicine, Massachusetts General Hospital, Harvard Medical School, Boston, Massachusetts, United States of America; University of Ottawa, Canada

## Abstract

**Background:**

There are sparse data on genetic, epigenetic and vitamin D exposure in African Americans (AA) with colon polyp. Consequently, we evaluated serum 25(OH) D levels, *vitamin D receptor* (*VDR*) polymorphisms and the methylation status of the tumor suppressor gene *dickkopf homolog 1* (*DKK1*) as risk factors for colon polyp in this population.

**Methods:**

The case-control study consisted of 93 patients with colon polyp (cases) and 187 healthy individuals (controls) at Howard University Hospital. Serum levels of 25(OH)D (including D3, D2, and total) were measured by liquid chromatography-mass spectrometry. DNA analysis focused on 49 single nucleotide polymorphisms (SNPs) in the *VDR* gene. Promoter methylation analysis of *DKK1* was also performed. The resulting data were processed in unadjusted and multivariable logistic regression analyses.

**Results:**

Cases and controls differed in vitamin D status (D_3_<50 nmol/L: Median of 35.5 in cases vs. 36.8 in controls nmol/L; P = 0.05). Low levels of 25(OH)D_3_ (<50 nmol/L) were observed in 86% of cases and 68% of controls and it was associated with higher risks of colon polyp (odds ratio of 2.7, 95% confidence interval 1.3–3.4). The SNP analysis showed no association between 46 *VDR* polymorphisms and colon polyp. The promoter of the *DKK1* gene was unmethylated in 96% of the samples.

**Conclusion:**

We found an inverse association between serum 25(OH)D_3_ and colon polyp in AAs. *VDR* SNPs and *DKK1* methylation were not associated with colon polyp. Vitamin D levels may in part explain the higher incidence of polyp in AAs.

## Introduction

Colorectal cancer is the third most common cancer in the United States [1]. It has higher incidence and mortality in African Americans (AA) than in other racial groups. Most colorectal cancers arise from adenomas, in a process described as the adenoma-carcinoma sequence [2], [3]. Like other cancers, initiation and progression of CRC are associated with accumulation of changes in genetic and epigenetic factors. Earlier studies demonstrated that CRC mortality is highest in populations that are least exposed to sunlight [4], [5]. As a consequence, they hypothesized that vitamin D, which is produced in the skin as a response to UV-B radiation, may have a protective role against colorectal cancer. Several studies have been done on the association of vitamin D and the development of colorectal cancer, which confirmed Garland and Garland's hypothesis [4], [5], [6]. Diets that are rich in lipids and low in calcium and vitamin D are strongly associated with high risk of colon cancer [6], [7]. Although the effects of vitamin D on the development of colon cancer have been extensively studied in whites [8], [9]there are few studies done in the African American population [10].

Studies have shown that diet and chemoprevention can modulate the progression of colon cancer [6]. Indeed, a diet of low calcium and vitamin D and elevated lipids can induce colonic tumors [7], [11]. However, these abnormal growths can be inhibited with supplements of calcium and vitamin D, hence demonstrating the crucial role played by the combination of these two elements in cancer prevention [12].

Studies have attempted to assess the effects of various single nucleotide polymorphisms (SNPs) of the vitamin D receptor (*VDR*) gene on the development of colon cancer. This gene is located on chromosome 12 and contains 9 exons [13]. The initial hypothesis is that variations in *VDR* expression or VDR protein sequence cause changes in levels of vitamin D. Since other studies suggest and here we confirm that vitamin D levels are associated with polyp or cancer risk, we test a composite hypothesis that variations in *VDR* are associated with polyp risk.

More than 470 SNPs in the *VDR* gene have been reported [14], [15], [16], [17], [18], [19], [20], [21]. Currently, the most extensively studied polymorphisms are the following: 1) rs10735810 or Fok1 in exon 2, 2) rs1544410 or Bsm1 in intron 8, 3) rs731236 or Taq1 in exon 9, 4) rs7975232 or Apa1 in intron 8, 5) rs757343 or Tru91 in intron 8, and 6) the poly (A) mononucleotide repeat in the 3′-UTR. The roles of the polymorphisms depend on their locations [18]. For example, Fok1 is within the DNA binding domain, near the 5′end, and the rest of the SNPs are in the 3′-UTR region within the ligand-binding domain. While the effect of most of these polymorphisms is unknown, the Fok1 polymorphism is known to lead to a T→C nucleotide substitution and a change in the start codon in exon 2 at the 5′ end of the *VDR* gene [22]. This new site (“f” allele) promotes the initiation of translation from the first codon instead of the second codon (“F” allele). The result is a protein that is 3 amino acids longer. The f allele product is a less potent transcriptional activator [23]. Conversely, the absence of Fok1 site results in a shorter VDR protein with higher transcriptional and biological activities [15]. Much remains to be elucidated with respect to the physiological effects of *VDR* polymorphisms.

One human gene that has been previously connected to both vitamin D levels and pathways involved in CRC is dickkopf homolog 1 (*DKK1*). The unusual gene name came about because orthologs of the human gene were discovered in lower organisms, the first one being the frog (*Xenopus)*. *DKK1* encodes a secreted protein involved in embryonic induction and in antagonizing the Wnt pathway [24]. Human DKK1 takes part in a host of complex cellular processes in different cell types. According to Pendas-Franco *et al* [24], this protein induces the proliferation of human adult bone marrow stem cells, inhibits osteoblastic differentiation, stimulates the differentiation of preadipocytes and inhibits the proliferation of crypt progenitor cells, induced by the transcriptional activity of the β-catenin/T cell factor complex in the small intestine and colon of mice. 1,25 (OH) D_3_ up-regulates the transcriptional activity of *DKK1*, thus connecting vitamin D levels to expression of a gene whose encoded protein function in an important pathway in the development of colorectal cancer. A common mechanism by which expression levels of genes are known to be inappropriately lowered in cancer is methylation of the promoter. Therefore, we evaluated whether *DKK1* is methylated unusually often in tumors from the cases used in our case-control study of vitamin D levels.

According to Davis et al. [15], unlike serum concentrations of 1,25(OH) D that are “tightly regulated and do not vary with geographic latitude or race”, serum concentrations of 25(OH)D are lower amongst persons with dark pigmentation and decrease as one moves away from the equator. Moreover, the melanin in people with darker complexion acts as a protective barrier against UV rays. As a consequence, vitamin D synthesis is considerably hindered [4], [5]. In addition to skin color and geographical location, vitamin D synthesis is also determined by age (the ability to produce vitamin D decreases with age), season of the year, fat malabsorption syndromes (e.g. cystic fibrosis), skin covering (sunscreen and clothing) and the level of air pollution [25]. Body mass index (BMI) is also associated inversely with the level of vitamin D in the serum. It is believed that vitamin D is sequestered in “subcutaneous fat” in obese individuals [26].

In this study, we suggest that the African American population treated at the Howard University Hospital (HUH, located in Washington, D.C.) is more prone to developing colon polyp, partly due to their vitamin D deficiency related to skin color, the geographic location and other unknown factors. The first objective of this study was to determine if the African Americans at HUH have a high frequency of vitamin D deficiency (defined as 25(OH)D<50 nmol/L) [5]. Secondly, was there an association between vitamin D deficiency and colon polyp and other variants such as sex, race, age, BMI, season of blood collection, alcohol and smoking status [27], [28], [29], [30]. Thirdly, the study assessed whether polymorphisms in the *VDR* gene are also polymorphic in the AA population at HUH and if so, whether any of them are associated with colon polyp. Lastly, we evaluated whether the DNA methylation status of the tumor suppressor gene *DKK1* is associated with the vitamin D deficiency and colorectal adenoma in AA.

## Materials and Methods

### Ethics Statement

This study was approved by the Howard University Institutional Review Board, and written, informed consent was obtained from all participants.

### Patients

The blood specimens were obtained based on two previous studies (between 2000 and 2004; 2008 and 2009) from African-American patients undergoing colonoscopy at Howard University Hospital. The total number of patients enrolled in this study was 280, including 93 cases (colon polyp) and 187 controls (healthy controls with no family history of CRC). Study participants were excluded if they any of these criteria: HIV infection, recent surgery or bone fracture, pregnancy or kidney disease. In both recruitment periods serum was isolated from fasting blood. We used the same criteria for case and controls. Samples were centrifuged for 10 minutes at a speed of 443×g at 4°C. Serum were stored at −20°C for later vitamin D quantitation. Both cases and controls were collected in the same time intervals, in the same clinic, and processed under the same protocol prior to analysis. The time from venipuncture to serum processing was approximately 2 hours. DNA was also isolated from whole blood samples using Qiagen FlexiGene Kit (Qiagen, Hilden, Germany).

### Vitamin D quantitation

250–500 µl aliquots of blood serum were shipped on dry ice to Massachusetts General Hospital (MGH) (Boston, MA) for serum 25 (OH) D measurements by liquid chromatography-mass spectrometry (LC-MS). This technique is replacing DiaSorin radioimmunoassay as the criterion standard [31]. The method involves the use of an isotope dilution: the deuterated stable isotope d3-25-hydroxyvitamin D. LC-MS assay was optimized in an MGH laboratory based on published procedures [32]. The limit of detection was 5 nmol/L for D_2_ and 7.5 nmol/L for D_3_. The between-run coefficient of variation (i.e. CV) for a quality control serum containing a total vitamin D concentration of 57 nmol/L was 7.5%. For all primary analyses, a prior categories were used based on clinically defined cut points introduced by Garland et al 2009 <50 nmol/L: insufficiency [5].

### Genotyping of *VDR* polymorphisms

To determine tagging SNPs, genotyped *VDR* polymorphisms in the Yoruba population were collected from public databases including Hapmap, dbSNP and Seattle SNPs. SNPs in the coding, non-coding , and 5′ and 3′ untranslated regions of *VDR* (46586959 to 46521297 Mb) were input into the program LDSelect [33] to determine tagging SNPs with minor allele frequencies>0.05 and r^2^>0.8. The allele frequency requirement was applied first to the public data to select some SNPs. That the public Yoruba samples have a high minor allele frequency does not imply that the minor allele frequency must be >0.05 in the HUH AA population. Previously tested *VDR* functional SNPs Taq1 and Fok1 were also genotyped. In total, we genotyped 57 *VDR* tagging SNPs were genotyped in this study. iPLEX assays were designed using the Sequenom (San Diego CA), Assay Design software. Primer sequences for PCR and single-base extension are available on request. Multiplex PCR was performed to amplify 5–10 ng of genomic DNA extracted from the surgical specimens. PCR reactions were treated with shrimp alkaline phosphatase (SAP) to neutralize unincorporated dNTPs. A post-PCR single-base extension reaction was performed for each multiplex reaction using concentrations of 0.625 µM for low-mass primers and 1.25 µM for high-mass primers. Reactions were diluted with 16 µL of H_2_O and fragments were purified with resin, spotted onto Sequenom SpectroCHIP microarrays and scanned by MALDI-TOF mass spectrometry. Individual SNP genotype calls were generated using Sequenom TYPE software which automatically calls allele-specific peaks according to their expected masses. Among the genotyped SNPs, 49 had minor allele frequencies >0.05 in the HUH AA population.

### Methylation analysis of *DKK1* gene

Methylation-specific quantitative real time PCR (Q-MSP) was used to quantify the amount of methylated DNA in the promoter of *DKK1*. DNA was isolated using the Qiagen, Germantown, MD) FlexiGene kit from whole blood samples. For each sample, 500 ng of DNA was modified using sodium bisulfite treatment (Zymo Research, Irvin CA), following the manufacturer's instructions. During this step, unmethylated cytosines, in CpG islands, are modified into uracils, while methylated cytosines remain the same. The immortalized cancer cell line SW48 and normal white blood cells were respectively used as positive and negative controls for DNA methylation. Real-time PCR was performed to amplify 3 ng of DNA modified with bisulfite. The real-time PCR ABI 7500 instrument (Applied biosystems Carlsbad, California) with a melting curve analyzing system was used. Each PCR reaction contained 12.5 µl of RT^2^ SYBR GREEN/ROX qPCR Master Mix (SA, Bioscience Corporation) with 0.5 µM of primers in a total volume of 20 µl. The fully methylated DNA extracted from SW48 cells was used as standard DNA for absolute quantification [34]. Primers were designed according to a published protocol [34], which also specified the PCR conditions and number of cycles.

### Statistical analysis

Serum concentrations of vitamin D in the study samples were reported as median (interquartile range). We used the natural log transformation for vitamin D concentration. Vitamin D levels and other continuous variables between cases (colon polyps) and normal controls were compared using the student's t-test. Categorical variables between cases and controls were compared with Chi-square tests. In a multiple logistic regression analysis the odds ratios (OR) of vitamin D levels on colon polyp were computed after adjusting for the effect of age, sex, season of sampling, BMI, smoking, and alcohol consumption. We also used the distribution of vitamin D in controls to calculate quartiles of serum 25-hydroxyvitamin D concentration and the effect of different serum vitamin D level on risk of the colon polyp. In the final analysis, a Bonferoni correction was applied to any nominally significant results. All analyses were performed by Stata 10.1 (StataCorp, College Station, TX). P values of 0.05 or lower were considered statistically significant. Tests of association between genotype and the polyp phenotype were done with the software PLINK [35].

## Results

### Association of vitamin D3 with risk of colon polyp

The possibilities of association of gender, alcohol, smoking and serum 25(OH)D level on the risk of developing colon polyp were examined in a cohort of 280 AA individuals. There were 93 cases with colon polyp and 187 normal colon controls. The colon polyps sample group consisted mainly of two types of neoplasms: tubular adenomas, which represented 76% of samples, and hyperplastic polyps, 16% of the case population. The remaining samples were cases of mixed polyps (tubular, tubovillous, and villous). The distribution of demographic variables and vitamin D concentration in cases and controls was determined (Table 1). Based on the data (Table 1), median vitamin D levels in cases and controls were 41.2 and 41.4 nmol/L, respectively. The median ages of cases and controls were 59 and 60 years, respectively. Frequency of current and past smoking was not different among the cases and controls but cases had higher frequency of current smoking (20% vs. 9% in controls, P = 0.01). Both cases and controls had deficiency of vitamin D, whose sources are diet and food supplements, as opposed to vitamin D_3_ that is also synthesized in the skin during sun exposure. Vitamin D_3_ concentration was lower in polyp cases (P = 0.05). By using the reference range (vitamin D levels 75–199 nmol/L as sufficient), both groups were deficient [36]. The median vitamin D_2_ concentration for cases and controls was 1.7 and 2.3 nmol/L (Table 1), respectively. For vitamin D_3_, the medians for patients and controls were 35.3 and 36.8 nmol/L, respectively, which shows Vitamin D deficiency. There were no significant differences between cases and controls on the following variables: age, body mass index, gender distribution, alcohol consumption, 25(OH)D_2_ and total Vitamin D concentration.

**Table 1:** Distibution of demographic variables and vitamin D concentration in cases (colon adenoma) and controls.

	Cases (n^1^ = 93)	Controls (n^1^ = 187)	P value
Age	59 (52–68)	60 (52–66)	0.68
Male, n (%)	49 (53%)	81 (44%)	0.15
Current or past smoker, n (%)	40 (44%)	63 (332%)	0.11
Current or past alcohol drinker n (%)	45 (50%)	97 (53%)	0.71
BMI (kg/m^2^)	28 (24–34)	29 (26–33)	0.66
D_2_ concentration (nmol/L)	1.7 (1.0–5.6)	2.3 (1.3–4.7)	0.34
D_3_ concentration (nmol/L)	35.3 (26.2–45.4)	36.8 (25.2–55.7)	0.05
Total Vit D concentration (nmol/L)	41.2 (30.2–51.8)	41.4 (29.4–65.6)	0.49

Results are in median (inter-quartile) unless otherwise indicated.

1 Number of cases (n = 4) and controls (n = 3): fluctuates (e.g. n = 90–93) due to missing value for some samples.

### Total 25(OH)D and its association with seasonal variation

**Figure 1:**
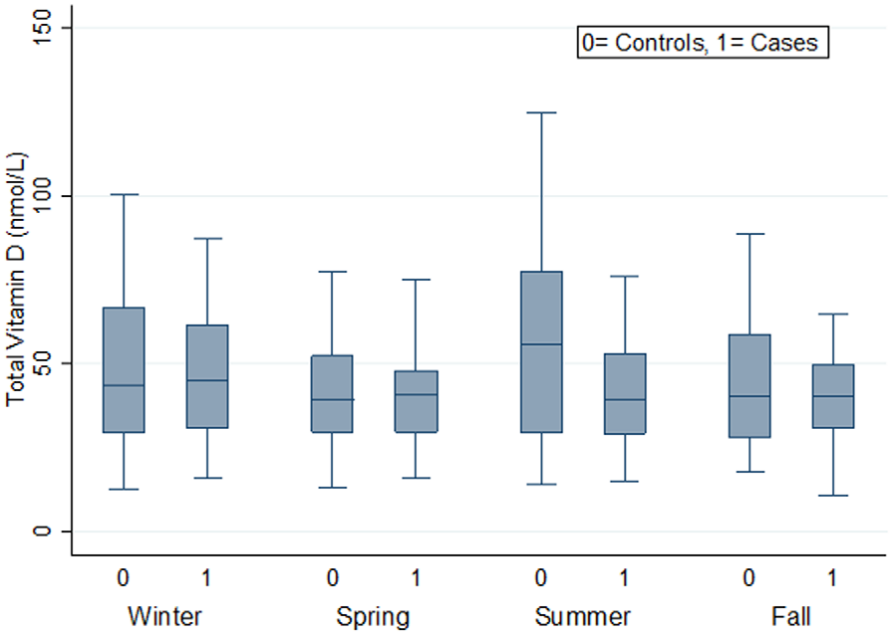
Total 25(OH)D and its association with seasonal variation. The concentration of total 25(OH)D in the colon polyp (n = 93) and normal (n = 187, healthy controls with no family history of CRC) samples across the seasons of the year was measured by liquid chromatography-mass spectrometry. Total 25(OH)D concentrations were marginally higher in winter and summer and were lower in spring and fall (P = 0.06).

The concentration of total 25(OH)D_3_ in the sample pool across the seasons of the year was measured (Figure 1). Total 25(OH)D concentrations were marginally higher in winter and summer and were lower in spring and fall (P = 0.06).

### Association between vitamin D concentrations and risk of colon polyp

Based on the range of vitamin D concentrations in controls, 4 different quartiles were generated. The odds ratios (OR) of different vitamin D concentrations on the risk of colon polyps was assessed (Table 2). No significant difference was found between the cases and controls, when looking at various concentrations of 25(OH)D_2_, 25(OH)D_3_ and total 25(OH)D. However, the results, point towards a general tendency of lower risk of polyps for the higher quartiles of total vitamin D and vitamin D_3_. The association of total 25(OH)D and 25(OH)D_3_ with the risk of colon polyp was analyzed (Table 3). Levels of 25(OH)D that are less than 50 (nmol/L) are in 83% of cases and only 69% of controls (P = 0.018). Similarly, 86% of cases and 68% of controls had insufficient 25(OH)D_3_ levels (P = 0.002). The latter difference remains significant (P = 0.007), after adjustment for age, sex, BMI, season, smoking, and alcohol usage. This remains significance even after Bonferoni correction. If the samples are partitioned by season, then spring (P = 0.07), summer (P = 0.07), and fall (P = 0.08) all show a similar trend towards significance, but winter (P = 0.9) does not. The comparisons for any specific season are not significant because the sample sizes become too small when the total sample set of 93 cases and 187 controls is partitioned by season. This indicates that lower levels of 25(OH)D_3_ and total 25(OH)D are associated with higher risks of colon polyp (with 50 nmol/L used as the threshold for deficiency).

**Table 2:** Odds ratio (95% CI) of different serum 25-hydroxyvitamin D concentration in risk of colon polyp.

	1^st^ quartile(n = 69)	2^nd^ quartile(n = 71)	3^rd^ quartile(n = 70)	4^th^ quartile(n = 70)	P for test of trend
25(OH)D_2_ (nmol/L)	<1.0	1.0–1.7	1.8–4.7	>4.7	
Unadjusted OR	1	0.9 (0.4–1.8)	0.5 (0.3–1.1)	0.8 (0.4–1.6)	0.3
Adjusted OR^1^	1	0.9 (0.4–2.0)	0.6 (0.3–1.2)	0.9 (0.4–1.9)	0.5
25(OH)D_3_ (nmol/L)	<25.3	25.3–35.9	36.0–50.4	>50.4	
Unadjusted OR	1	1.5 (0.8–3.1)	1.9 (0.9–3.8)	0.5 (0.2–1.2)	0.2
Adjusted OR^1^	1	1.5 (0.7–3.2)	1.8 (0.9–3.9)	0.6 (0.2–1.3)	0.3
25(OH)D total (nmol/L)	<29.5	29.5–41.2	41.3–57.7	>57.7	
Unadjusted OR	1	1.4 (0.7–2.8)	2.0 (1.0–4.0)	0.5 (0.2–1.1)	0.3
Adjusted OR^1^	1	1.4 (0.7–3.1)	1.9 (0.9–4.1)	0.6 (0.3–1.4)	0.4

1 Adjusted for effect of age, sex, BMI, season, smoking and alcohol usage.

**Table 3:** Odds ratio (95% CI) of insufficient vitamin D status in risk of colon polyp.

	Cases(N = 92)	Controls(N = 186)	Unadjusted		Adjusted^1^	
			OR (95% CI)	P value	OR (95% CI)	P value
Total 25(OH)D <50.0, no (%) (nmol/L)	76 (83)	128 (69)	2.1 (1.1–3.9)	0.018	1.7 (0.9–3.4)	0.10
25(OH)D3 <50.0, no (%) (nmol/L)	79 (86)	127 (68)	2.8 (1.5–5.5)	0.002*	2.6 (1.3–5.2)	0.007*

1 Adjusted for effect of age, sex, BMI, season, smoking and alcohol usage.

*Significant after Bonferoni correction.

### DNA Methylation of *DKK1* promoter is not associated with the risk of colon polyp

DNA methylation in the *DKK1* promoter was quantified using the Q-MSP method in 280 samples. In this pool of samples, the *DKK1* promoter was not methylated. There were 62, 65 and 3 patients with <1%, 1–10%, and >10% *DKK1* methylation in control vs. 37, 31, and 1 patients in cases, respectively. Out of 93 colon polyp samples, only 3 individuals showed methylation level higher than 20%.

### *VDR* SNPs not associated with risk of colon polyp

Forty-nine of the 57 *VDR* SNPs genotyped had a minor allele frequency >0.05 in the study population (supporting information Table S1). No significant association was found between the SNPs and the risk of colon polyp in the patients' pool. Moreover, the allele frequencies of each SNP were similar in cases and controls. Some studies done subsequent to our choice of SNPs indicate that other SNPs in the *VDR* gene are polymorphic in African Americans [37], [38], [39]. These additional SNPs are sufficiently close to at least some of the SNPs we genotyped and hence would be expected to be in strong linkage disequilibrium with SNPs we genotyped.

## Discussion

Prior studies have shown that the level of serum 25(OH)D is inversely correlated with the rate of colorectal cancer among white Americans [5], [6], [40], [41]. In the present study, we demonstrated that serum total 25(OH)D and 25(OH)D_3_ concentrations among African Americans patients at Howard University Hospital (HUH) are inversely associated with the risk of colon polyps. An insufficient amount of vitamin D (<50 nmol/L) associates with higher chances of developing colon polyps [5]; Chan, 2010 #47}. According to the reference range, vitamin D levels <50 nmol/L in the HUH sample group are on the lower end or insufficient. These results can be explained by the fact that today's lifestyle requires most people to work indoors. Consequently, if people do not expose themselves to the sun daily, and do not fortify their diets or supplement with vitamins, they run the risk of vitamin D deficiency. This is likely what happened in many AA individuals enrolled in this study [42], [43], [44]. Moreover, the darker complexion of AA individuals may exacerbate this deficiency because of the melanin screen effect [4], [5], [40], [41]. Other studies showed that diets and chemoprevention can modulate the progression of colon cancer [6]. Indeed, a diet of low calcium and vitamin D and elevated lipids can induce colonic tumors [7], [11]. Although the effects of vitamin D on the development of colon cancer have been extensively studied in white Americans [8], [9], there are sparse data from the African American population [10] to determine the levels of 25-hydoxyvitamin D (25[OH]D) in the serum of healthy vs. ill individuals.

Earlier studies demonstrated that cancer mortality had the highest rate in the populations that were least exposed to sunlight [4], [5]. As a result, they hypothesized that vitamin D, which is produced in the skin as a response to UV-B radiation, may have a protective role against colorectal cancer. Since then, several studies have been done on the association of vitamin D and the development of colorectal cancer, which confirmed Garland and Garland's hypothesis [4], [5], [6]. It is not clear why total 25(OH)D concentrations were a bit higher in winter, even though this level was higher in summer and lower in spring and fall. AA may have different environmental exposures including diet that might change the composition and the activities of the intestinal microbiota, in turn affecting fecal genotoxicity/mutagenicity that is thought to be associated with carcinogenesis. Mai et al reported an increased intake of heterocyclic amines (HCA) and a decreased dietary intake of vitamins including vitamin D (p<0.05) in AA that correlated with differences in fecal microbiota composition [10]. Dietary habits of AA, including increased HCA intake and decreased vitamin D intake might at least partially contribute to CRC through modifications of gut microbiota composition that result in changes of the intestinal milieu [10]. In the human body, vitamin D_3_ is needed for many physiological activities. Consequently, it is found in larger concentrations than vitamin D_2._ Indeed, Houghton *et al*. cite three factors explaining the “better bio-efficacy” of vitamin D_3_: more affinity to vitamin D binding proteins, small variation in chemical structure and hepatic hydroxylases [45]. The D_2_ form has been distinguished from the D_3_ form in various epidemiological studies [27]. Measurement of serum vitamin D may have some degree of errors. A CV of 7.5% in our assay indicates a mild imprecision. This may cause some noise to our relationships and especially decrease our power to find significant relationships.

The strengths of our study include the uniqueness of our population and that we are basing our comparisons on serum Vitamin D while other studies in the field rely on dietary Vitamin D intake as deduced from diet questionnaires. One weakness is the small sample size. To obtain significant results for D_2_ and D_3_, we would need a much larger sample (>1000 subjects) in each group for the calculations in Table 1, We are pursuing this goal with a more comprehensive analysis in the near future. At the moment, our calculations show that the value for Vitamin D2 and total Vitamin D are not significantly different between the two groups in our population. We are joining efforts with other centers with interest in colon cancer in African Americans to have samples from other areas of the United States for our future studies.

With respect to promoter methylation of the tumor suppressor gene *DKK1* and 46 SNPs in the *VDR* locus, no association was found with risk of colon cancer. We found there are no associations between the methylation and Vitamin D consistent with Pendás-Franco study [24]; as seen in Europeans, *DKK1* methylation in African Americans is not directly associated with vitamin D.

According to Davis *et al*, over 470 VDR SNPs are known [15]. Their distribution and frequency vary among ethnic groups. Most of the work done on VDR polymorphisms has been conducted in Caucasian populations and has focused on the six SNPs mentioned in the introduction. Two of them, Fok1 and Taq1, have been associated with breast cancer risk [14]. However, Barroso *et al* [14] emphasized that the results of their study should be replicated on a larger samples. In our study, Taq1 had no significant association with risk of colon polyp; Fok1 was genotyped but had too low a minor allele frequency in our study population to be tested for association. Moreover, none of the comprehensive tagging SNPs in *VDR* were significantly associated with colonic polyps. Diet could be a modifier factor in the *VDR* analysis, but we did not have complete diet information on the current patients.

Causal inference from a case-control study should be drawn with extreme caution. Case-control studies are prone to a range of bias, including selection bias, confounding bias and lack of temporality. In this study, we tried to reduce the effect of confounding variables by multivariate analysis. The potential sources of selection bias were reduced by selecting the patients and controls from same race and institute, and especially the same clinic.

In sum, we found an inverse association between serum 25(OH)D3 and colon polyp in AA. *VDR* SNPs and *DKK1* methylation were not associated with colon polyp. The novelty of this study is due to its unique population and their high risk of CRC. Since pigment has been associated with vitamin D levels and vitamin D levels have been associated with CRC, studies focused on an AA population are especially needed to clarify these relationships. Overall, the low Vitamin D levels in AA may in part explain the higher incidence of polyp in AA. Larger studies of the AA community involving more of the genetic elements involved in the Vitamin D pathway/metabolism are needed to shed more light on the Vitamin D implication in colonic oncogenesis in this population.

## Supporting Information

Table S1 (PDF)
