# Supplementary material for: Case-Control Study of Vitamin D, dickkopf homolog 1 (DKK1) Gene Methylation, VDR Gene Polymorphism and the Risk of Colon Adenoma in African Americans
Source: PLoS One. 2011 Oct 13;6(10):e25314. doi: 10.1371/journal.pone.0025314 (PMC3192764; doi:10.1371/journal.pone.0025314)
Supplement: Table S1 — (PDF) [file pone.0025314.s001.pdf]

Table 4– PLINK results of tests of association of 49 SNPs in the *VDR* gene, which is located on chromosome 12, with affection (colon polyp) status. No SNPs are nominally associated with affection status. MAF(aff) and MAF(un) are the minor allele frequencies in cases (affected) and controls (unaffected), respectively. The CHISQ (1df) , P-value and OR(odds ratio) are as reported by PLINK. Two SNPs we genotyped are known by other names: Taq1 is used to identify rs73126; Fok1 is used to identify rs10735810.

| CHR | dbSNP id | Position(bp) | MAF(aff) | MAF(un) | CHISQ | P | OR |
|---|---|---|---|---|---|---|---|
| 12 | rs11574141 | 46521297 | 0.07212 | 0.07035 | 0.006445 | 0.936 | 1.027 |
| 12 | rs2853563 | 46522005 | 0.1875 | 0.154 | 1.105 | 0.2931 | 1.267 |
| 12 | rs9729 | 46522890 | 0.41 | 0.4005 | 0.04897 | 0.8249 | 1.04 |
| 12 | rs7954412 | 46523554 | 0.101 | 0.0825 | 0.5763 | 0.4478 | 1.249 |
| 12 | rs739837 | 46524488 | 0.4709 | 0.4391 | 0.5523 | 0.4574 | 1.137 |
| 12 | rs731236 | 46525024 | 0.2794 | 0.2739 | 0.02075 | 0.8855 | 1.028 |
| 12 | rs11574114 | 46525150 | 0.1731 | 0.1457 | 0.7796 | 0.3773 | 1.227 |
| 12 | rs12314197 | 46528989 | 0.2308 | 0.1919 | 1.262 | 0.2614 | 1.263 |
| 12 | rs7962898 | 46529104 | 0.3382 | 0.3706 | 0.6098 | 0.4348 | 0.8682 |
| 12 | rs7967152 | 46530451 | 0.4175 | 0.4036 | 0.1085 | 0.7418 | 1.059 |
| 12 | rs2239185 | 46530826 | 0.476 | 0.4246 | 1.46 | 0.227 | 1.231 |
| 12 | rs7971418 | 46531502 | 0.4903 | 0.4419 | 1.277 | 0.2584 | 1.215 |
| 12 | rs7975128 | 46532095 | 0.2621 | 0.2702 | 0.045 | 0.832 | 0.9595 |
| 12 | rs11168264 | 46533130 | 0.1942 | 0.2323 | 1.152 | 0.2831 | 0.7962 |
| 12 | rs7305032 | 46536127 | 0.2788 | 0.2814 | 0.004439 | 0.9469 | 0.9874 |
| 12 | rs11168266 | 46537800 | 0.4804 | 0.4442 | 0.7111 | 0.3991 | 1.157 |
| 12 | rs11168267 | 46537809 | 0.07692 | 0.08794 | 0.2151 | 0.6428 | 0.8643 |
| 12 | rs11168268 | 46538079 | 0.3846 | 0.3693 | 0.1359 | 0.7123 | 1.067 |
| 12 | rs12308082 | 46538406 | 0.08173 | 0.07071 | 0.2403 | 0.624 | 1.17 |
| 12 | rs2248098 | 46539623 | 0.4563 | 0.505 | 1.289 | 0.2562 | 0.8226 |
| 12 | rs987949 | 46540943 | 0.233 | 0.2739 | 1.178 | 0.2778 | 0.8055 |
| 12 | rs2239182 | 46541678 | 0.4135 | 0.4121 | 0.001107 | 0.9735 | 1.006 |
| 12 | rs2107301 | 46541837 | 0.1538 | 0.1683 | 0.21 | 0.6468 | 0.8982 |
| 12 | rs1540339 | 46543593 | 0.1779 | 0.2222 | 1.633 | 0.2012 | 0.7573 |
| 12 | rs2239179 | 46544033 | 0.3627 | 0.3568 | 0.02067 | 0.8857 | 1.026 |
| 12 | rs11574070 | 46544162 | 0.101 | 0.07323 | 1.381 | 0.2399 | 1.421 |

| | | | | | | | |
|---|---|---|---|---|---|---|---|
| 12 | rs12717991 | 46545393 | 0.2837 | 0.309 | 0.4191 | 0.5174 | 0.8853 |
| 12 | rs886441 | 46549231 | 0.4126 | 0.3975 | 0.1293 | 0.7192 | 1.065 |
| 12 | rs2189480 | 46550095 | 0.3107 | 0.3434 | 0.6553 | 0.4182 | 0.8616 |
| 12 | rs3819545 | 46551273 | 0.1971 | 0.2525 | 2.34 | 0.1261 | 0.7267 |
| 12 | rs3782905 | 46552434 | 0.2573 | 0.215 | 1.375 | 0.2409 | 1.265 |
| 12 | rs11574050 | 46558728 | 0.101 | 0.0825 | 0.5763 | 0.4478 | 1.249 |
| 12 | rs10783218 | 46559010 | 0.1845 | 0.1742 | 0.09691 | 0.7556 | 1.072 |
| 12 | rs10735810 | 46559162 | 0.226 | 0.2161 | 0.07786 | 0.7802 | 1.059 |
| 12 | rs2408876 | 46559832 | 0.5049 | 0.4675 | 0.7601 | 0.3833 | 1.161 |
| 12 | rs2254210 | 46559981 | 0.3269 | 0.345 | 0.1996 | 0.655 | 0.9222 |
| 12 | rs11574044 | 46562101 | 0.2427 | 0.2613 | 0.2469 | 0.6193 | 0.9061 |
| 12 | rs11574041 | 46562488 | 0.07212 | 0.0775 | 0.05673 | 0.8117 | 0.9251 |
| 12 | rs2238136 | 46563980 | 0.06731 | 0.06 | 0.1247 | 0.724 | 1.131 |
| 12 | rs2238135 | 46564457 | 0.3365 | 0.3191 | 0.1894 | 0.6634 | 1.082 |
| 12 | rs2853564 | 46564754 | 0.1394 | 0.1439 | 0.02278 | 0.88 | 0.9635 |
| 12 | rs4760648 | 46566932 | 0.5098 | 0.485 | 0.3325 | 0.5642 | 1.104 |
| 12 | rs2853559 | 46569072 | 0.1827 | 0.1975 | 0.1931 | 0.6604 | 0.9083 |
| 12 | rs11168287 | 46571681 | 0.2981 | 0.2675 | 0.6375 | 0.4246 | 1.163 |
| 12 | rs4328262 | 46571915 | 0.3798 | 0.315 | 2.572 | 0.1088 | 1.332 |
| 12 | rs4334089 | 46572282 | 0.3835 | 0.3662 | 0.1743 | 0.6764 | 1.077 |
| 12 | rs3890733 | 46575640 | 0.1422 | 0.1667 | 0.6069 | 0.4359 | 0.8286 |
| 12 | rs7302235 | 46579105 | 0.5 | 0.4899 | 0.0552 | 0.8142 | 1.041 |
| 12 | rs7136534 | 46580893 | 0.1106 | 0.09296 | 0.475 | 0.4907 | 1.213 |
